# Arthroscopic Treatment of Mild/Borderline Hip Dysplasia with Concomitant Femoroacetabular Impingement—Literature Review

**DOI:** 10.1007/s12178-022-09765-4

**Published:** 2022-06-16

**Authors:** Ran Atzmon, Marc R Safran

**Affiliations:** Department of Orthopaedics Surgery, Stanford University, 450 Broadway, Redwood City, CA 94063 USA

**Keywords:** Borderline hip dysplasia (BDH), Femoroacetabular impingement (FAI), Hip arthroscopy, Hip microinstability, Periacetabular osteotomy (PAO)

## Abstract

**Purpose of Review:**

This literature review aims to survey the current knowledge about the management FAI in the setting of borderline hip dysplasia.

**Recent Findings:**

With better understanding, hip arthroscopy has recently been advocated for treating mild or borderline hip dysplasia (BDH) with concomitant femoroacetabular impingement (FAI) despite early studies that condemned its use. Recent outcome data have demonstrated that hip arthroscopy is a viable option in BDH, with and without FAI, and has been gaining wider acceptance. Hip arthroscopy can address the concomitant soft tissue and bony intra-articular pathologies and obviate the necessity for other surgeries. Moreover, hip arthroscopy may be used as an adjuvant treatment to other procedures such as a periacetabular osteotomy (PAO).

**Summary:**

Hip arthroscopy for BDH is an evolving procedure with promising short- and mid-term outcomes. The combination of BDH and FAI is becoming recognized as a problem in its own right, requiring dedicated treatment.

## Introduction

In the twenty-first century, hip arthroscopy has gained popularity, becoming the mainstay diagnostic and treatment tool for the majority of non-arthritic intra-articular hip pathologies. Nonetheless, there is still an ongoing debate regarding the exact definition, utilization, and effectiveness of hip arthroscopy in borderline hip dysplasia [1•, 2, 3•, 4–6]. The osseous structure of the hip joint is mainly comprised of the bony acetabulum and the femoral head and neck, with the acetabular architecture providing the majority of the hip osseous stability at the expense of range of motion. Theoretically, the femoroacetabular joint congruency lessens the importance of the soft tissue constraints in hip biomechanics. Abnormalities in the osseous structure may influence the joint congruency and inherent stability by changing the force distribution and contact area at the joint surface and manifest as hip micro- or macro-instability [1•, 5, 7•, 8, 9, 10••]. The most common pathology that represents and encompasses most of these bony abnormalities is developmental dysplasia of the hip (DDH), which is often seen with an abnormal acetabular orientation influencing its coverage, inclination, depth, and pathological femoral neck version.

### Definition of Dysplasia and Borderline Dysplasia

Various measurements have been suggested to appraise the severity of hip dysplasia; among them (and currently most commonly used) is the lateral center-edge angle (LCEA) of Wiberg [11] which measures the acetabular depth on an anteroposterior (AP) pelvic radiograph. This angle consists of a line from the center of the femoral head straight up, and another from the center of the femoral head to the lateral edge of the sourcil (not necessarily the most lateral aspect of the acetabulum). A normal angle is considered between 25 and 39°, with an LCEA<20° implicating a dysplastic hip. An angle between 20 and 25° is commonly defined as borderline hip dysplasia (BHD), with some studies defining it between 18 and 25° [1•, 3•].

The anterior center-edge angle (ACEA) which is measured on a false profile radiograph assesses the anterior acetabular coverage. This is an angle comprised of a line from the center of the femoral head straight up, and another from the center of the femoral head to the anterior sourcil. The angle was first described by Lequesne and de Seze in 1961 and presented normal values between 20 and 45°, with an ACEA less than 20° signifying hip dysplasia [1•, 12].

The Tönnis angle or acetabular roof angle is also measured on an AP pelvic radiograph and represents the weight-bearing surface of the acetabulum (i.e., acetabular sourcil), and its inclination and acetabular index (AI). An angle between 0 and 10° is considered normal, whereas an angle >10° signifies hip dysplasia and structural instability [1•, 13].

Though the LCEA is widely accepted to identify hip dysplasia and lateral coverage of the femoral head, this measurement may not be enough in borderline dysplasia with painful hip [14]. Hip dysplasia is a 3-dimensional problem that affects the lateral femoral head coverage/lateral acetabular extension, anterior coverage, acetabular and femoral version, and even neck-shaft angulation.

Vahedi and colleagues [15] noticed that occasionally painful hips might show normal radiographic characteristics even when the femoral head coverage is compromised. They calculated the coverage index (CI), which reflects the acetabular volume and discovered that the mean CI was significantly greater in the non-dysplastic group compared with the low-volume dysplastic cohort. Siebenrock et al. [16] introduced the anterior wall index (AWI) and posterior wall index (PWI), to quantify the anterior and posterior wall femoral head coverage in patients with hip pain. The authors concluded that these measurements could be used as supplemental data for radiographic analysis. McClincy et al. [14] identified patients with borderline dysplasia with an LCEA between 18 and 25°, in addition to other relevant radiographic measurements. The authors concluded that solely using the LCEA is insufficient to predict which surgical procedure should be performed and recommended using ACEA and AWI as independent measures to guide the treatment toward hip arthroscopy or PAO. Several criteria were defined to determine the acetabular version which is directly correlated to hip pathologies, such as posterior wall sign, crossover sign, and ischial spine sign.

Normal acetabular version is considered between 13 and 20° anteriorly, with excessive anteversion usually seen with DDH and retroversion in pincer-type FAI [17–19]. Dysplastic hips are usually present with alterations in the acetabular and femoral versions [20]. The exact number of patients presenting with BDH and concomitant FAI due to cranial retroversion is still unclear, though there is a growing number of studies identifying patients suffering from these two pathologies who were treated arthroscopically with hip preservation surgery [3•, 5•, 21–24]. Furthermore, it has been shown that cam deformity in the presence of BDH tended to be distributed across the proximal region of the femoral neck rather than the distal regions [25].

### Borderline Dysplasia with CAM Morphology—Instability vs Impingement

Beck and colleagues [26••] recognized that not all patients with acetabular dysplasia had hip instability. They noted a significant number of patients with coxa magna or more frequently, Cam FAI, that is to say, loss of femoral head-neck offset. These patients did not have instability but impingement. The authors sought to identify a radiographic measure to differentiate which patients with BDH had microinstability or loss of acetabular contact as opposed to impingement. Beck, Wyatt, and associates [26••] found the Femoro-Epiphyseal Acetabular Roof (FEAR) index as a way to differentiate the 2 different causes of pain in borderline dysplastic patients. The FEAR index is the angle formed between a line extending from the femoral head physeal scar and a line representing the acetabular roof (the line connecting the medial aspect of the sourcil at the cotyloid fossa and the lateral edge of the acetabulum). The authors compared the FEAR index to the LCEA and acetabular index (AI), with respect to intra- and inter-observer reliability and concluded that in the setting of LCEA <25° and FEAR index of <5°, the hip is considered stable [26••]. Batailler et al. [27] concluded that the cutoff value of 2° can predict hip stability with 90% probability. Truntzer and colleagues [28] further validated the FEAR index and concluded that it can be utilized to identify unstable hips in both BDH as well as in hips that do not have dysplastic features.

## BDH and Intra-articular Pathologies

In hip dysplasia, the contact area between the acetabulum and femoral head is reduced and concentrated on a small zone on the lateral aspect of the acetabulum. According to previous studies, the contact load and force transmitted in this area can reach 260% of the body weight in the single-leg stance [29, 30]. Moreover, the normal hip center of rotation changes and becomes more posterosuperior, leading to abnormal pressure at the labrum and the articular cartilage [31]. Several studies have found an association between hip dysplasia and labral tears, especially in the anterior part of the labrum [3•, 10••, 32]. According to some investigators, the mean prevalence for labral tear can reach 77% [3•, 4, 5•]. Further, these publications identified that at the tear area, the labrum became hypertrophied leading to its impingement between the acetabulum and femoral head, which may explain mechanical symptoms often seen in this population, such as pain, locking sensations, and clicking, (though some of this may be due to iliopsoas snapping) [3•, 10••, 32].

Due to the acetabular undercoverage in BDH, the labrum has to adapt in size and length to compensate for the diminished articular bony socket and create a total osseolabral coverage equal to the non-dysplastic hip [3•, 32, 33]. Labral injury has been linked to chondral lesions in up to 72% of cases identified at hip arthroscopy. Due to the lack of sufficient osseous coverage in DDH, the acetabular labrum expands and hypertrophies superiorly to contain the incongruent joint, making it redundant [34••]. It has been suggested that in dysplastic situations, the labrum participates in the abnormal load transfer at the hip joint, causing it to become hypertrophied to withstand these forces. The labrum, being soft tissue, can break down over time due to these increased load-bearing forces [34••].

On the other hand, this adaptive hypertrophy can also be related to incomplete ossification of the cartilaginous acetabulum during hip development [20, 33, 35]. Cartilage hypertrophy is also seen in dysplasia. In patients with borderline and frank dysplasia, Ashwell et al. [36•] found an increased cartilage thickness at the lateral sourcil of the acetabulum (i.e., the weight-bearing zone). The authors concluded that this cartilaginous thickness could indicate a compensatory reaction to the lack of bony coverage and might serve as an instability marker [36•].

Both hip dysplasia and FAI may result in labral tears and acetabular chondral flaps, though the morphological presentation of the chondral damage and the mechanism leading to it vary significantly [37]. Previous studies have demonstrated profound structural alterations such as hypertrophy and increased thickness of the articular cartilage in the face of dysplasia, and exposure of the cartilage to a non-contained area [1•, 3•, 20, 32, 36•], presumably as a result of compensatory mechanism to the shear stress acting on the cartilage during weight-bearing and biomechanical changes occurring in the joint. These may further develop into an “inside-out” type articular cartilage flap tear, where a central articular defect creates a chondrolabral sleeve that propagates peripherally [3•, 10••, 33, 37]. Alternatively, the excessive femoral head motion within the acetabulum may result in edge loading of the shallow acetabulum, resulting in labral chondral separation and an inside-out wear pattern of the acetabular rim (without flap) due to the femoral head subluxing on the edge of the acetabular rim [3•, 10••, 33, 37]. In contrast, FAI results in impingement between the non-spherical femoral head and the acetabular rim (i.e., cam-type), or acetabular overcoverage which is jammed into the femoral head-neck junction (i.e., pincer-type) demonstrates different labral pathologies. In cam-type impingement, the compression forces created by impingement act on the labral-cartilaginous junction, thus initially sparing the labrum [10••, 38, 39, 40•], but abutting the acetabular articular cartilage, causing the “carpet delamination” phenomenon. In pincer-type impingement, the hip range of motion is restricted due to the acetabular overcoverage, causing the labrum to impinge and become trapped between the femoral head-neck junction and acetabular rim. This may lead to subsequent subluxation of the femoral head and posteroinferior cartilage abrasion [39, 40•]. Additionally, due to the pincer mechanics, the acetabular rim is also compressed, causing smaller outside-in delamination of the acetabular cartilage. In both cam- and pincer-type impingement, the chondral and labral injury usually starts at the outer edge of the joint and progresses centrally creating an “outside-in” type articular cartilage flap tear [3•, 10••, 40•, 41]. Bolia et al. [42] showed that patients with FAI and BDH are more prone to suffer from advanced chondral damage (Outerbridge grades III and IV), with significantly larger chondral defects on the acetabular surface than patients with non-borderline dysplastic hips. Other studies also found a strong correlation between BDH and high-grade femoral head cartilage injury [21, 22, 43]. Kaya et al. [43] reported full-thickness articular defects extending as far as the posterior-superior zone at the femoral head. Moreover, the authors found the incidence of full-thickness cartilage damage to be high in patients exhibiting FAI and BDH compared to those with joint laxity and acetabular dysplasia [43]. These findings are further supported by other studies which examined the combination of BDH with FAI [21, 22].

The ligamentum teres (LT) also play an important role in hip biomechanics and range of motion. It was shown that arthroscopically cutting the LT results in hip instability, particularly in hip rotational movement, and especially at greater than 90° of hip flexion [44–46]. The LT creates a “ball and string” effect by spiraling around the femoral head and preventing it from subluxation in extreme movements by becoming taut [44–46]. Additionally, this effect actively maintains the femoral head in the acetabular socket, thus augmenting the hips’ terminal range of motion. In hip dysplasia, where the bony coverage is insufficient, and the hip capsule becomes redundant or lax, the LT becomes more important and assumes a primary role in controlling the hip motion [47, 48]. Ligamentum teres rupture is often seen in hip microinstability, particularly in hip dysplasia patients [49–51].

If not recognized and treated in time, these intra-articular pathologies may further develop to create a vicious cycle resulting in hip instability. Hip instability is commonly defined as extra physiologic hip motion that causes pain with or without symptoms of hip joint unsteadiness [7–9]. As discussed, the alterations in the osseous structure of the hip joint are frequently seen in dysplastic conditions, where the contact area between the acetabulum and femoral head is reduced and concentrated on a small zone on the lateral aspect of the acetabulum. Moreover, the normal hip center of rotation changes in hip dysplasia and becomes more posterosuperior, leading to abnormal pressure at the labrum and the articular cartilage [5, 8, 10••, 33, 36•]. In FAI, the osseous impingement during an extreme hip range of motion can cause the femoral head and neck to abut against the acetabular rim, thus leading to damage from impingement as well as creating a lever arm acting on the femoral head and subluxating it out of the acetabular socket, leading to instability [7•, 9].

## Arthroscopic Treatment

The goals of surgery are to address the soft tissue pathologies such as labral tears, articular cartilage damage, capsular laxity, including iliofemoral and ischiofemoral ligaments, and in some situations, the bony abnormalities. The surgical treatment option for BDH with or without FAI may vary from hip preservation surgery such as hip arthroscopy and acetabular reorientation (i.e., periacetabular osteotomy (PAO)), to hip replacement. It has been generally accepted that hip preservation surgeries, such as arthroscopy and/or PAO, are contraindicated in the setting of significant arthritis. It is also generally accepted that significant hip dysplasia is best treated with an open acetabular +/− femoral procedure, such as a PAO or femoral osteotomy, as that is the way to treat the underlying pathology (Bony malformation), though frequently ignoring the labral pathology. This has been supported by early studies of hip arthroscopic treatment of labral pathology in the setting of hip dysplasia, demonstrating generally poor outcomes, as the bony problem(s) was not addressed [52–56]. The exception to this is the arthroscopic-assisted shelf procedure described by Uchida [57], in which data is sparse. In the last decade, hip arthroscopy has gained popularity as the treatment of choice for mild degrees of hip dysplasia, borderline dysplasia. Due to the evolving nature of this field, long-term outcomes of arthroscopic treatment for BDH are yet to be reported. However, short-term, and now even some medium-term, outcomes evaluating the Patient-Reported Outcome Measures (PROs) look promising, showing significant improvement in Modified Harris Hip Score (mHHS) as well as other scoring such as the average Hip Outcome Score-Sport-Specific scale (HOS-SSS), visual analog scale (VAS), and Non-Arthritic Hip Score (NAHS). Most of the aforementioned studies measured an average of 20 points or more improvement in the Modified Harris Hip Score with hip arthroscopy. Nawabi et al. [24] studied patients suffering from borderline dysplasia and FAI who were treated arthroscopically and reported more than 20 points of mean improvement in the mHHS after arthroscopic intervention (preoperative 61.7 to postoperative 86.2), and even greater improvement in the HOS-ADL score and the HOS-SSS (76.0 to 93.2 and 54.6 to 85.4, respectively) [24]. Domb et al. [58–61] followed a cohort of patients with BDH and concomitant intra-articular pathologies who underwent arthroscopic labral surgery with capsular plication. The patients were evaluated preoperatively and at 1, 2, and 5 years postoperatively. At 2-year follow-up, all PROs showed a significant improvement compared with the preoperative evaluation, with 20.7, 17.5, 27.6, and 20.0 points improvement in the mHHS, HOS-ADL, HOS-SSS, and NAHS respectively. The VAS was also reduced by 3.16, and only 6 patients (11%) underwent revision surgery unrelated to the original operation. At the 5-year follow-up, the postoperative improvement was maintained, demonstrating significant improvements with 15.6, 18.7, and 19.0 points gains in the mHHS, HOS-SSS, and NAHS respectively, and VAS decrease of 3.8 points. The revision rate was 19%, followed by improved PRO scores. Though it can be inferred that an additional 14% of hips did not meet the 70-point threshold for the mHHS, thus having a clinical failure without revision surgery, comprising an overall rate of 33% of inadequate clinical results [59, 62]. Of note, out of the original 55 cases included in the 2-year follow-up, only twenty-five hips (24 patients) met the 5-year inclusion criteria. These findings demonstrating more than 50% reduction may pose a bias, which can influence the latter results [58, 59, 62, 63]

As previously described, the labrum and the articular cartilage undergo metaplasia as a result of the forces of BDH and instability, attempting to compensate for the acetabular undercoverage by becoming hypertrophied and more thickened respectively [1•, 3•, 20, 32, 36•]. Nonetheless, other studies such as by Landa et al. [64] attributed the hypertrophied ridge of fibrocartilage in the superolateral region of the acetabulum to the pressure caused by the displaced hip on this region. They also speculated that this fibrocartilaginous overgrowth might prevent the concentric reduction of the dysplastic hip [64].

During the decision-making process, the intraoperative appearance of the labrum should be evaluated. Generally speaking, the goal is to retain the labrum whenever possible for its many functions, including joint stability. If the labrum cannot be repaired, consideration should be given to labral reconstruction [65, 66]. Several indications for labral reconstruction in the face of BDH have been published, including poor or nonviable tissue quality, severely torn labrum, no evidence of severe chondral damage on preoperative imaging, and no evidence of advanced osteoarthritis [66–68]. Prior to labral repair or reconstruction, the underlying acetabular rim should be decorticated to promote labral healing, and the labrum itself should be trimmed to a normal size if repaired. Caution must be exercised not to over-decorticate the acetabular rim, which may increase the dysplasia, and potentially increase the risk or degree of instability [4, 7•, 9, 66]. Chandrasekaran et al. [58] have discussed the recommended amount of rim recession and defined a 2-mm threshold as the maximal value, beyond which hip instability may occur. Philippon et al. [69, 70] devised a formula that integrated the change in degrees of the LCEA and the amount of acetabular rim resection. According to this formula, a 2-mm reduction of the acetabular rim may result in a 3° change of LCEA [3•]. Hence, in the presence of hip dysplasia, acetabular rim resection should be minimized to prevent further hip instability.

Graft choice for labral reconstruction may include allograft or autograft substitutes, with the latter being further divided into remote or local autograft such as the hamstring and iliotibial band (ITB), or the indirect head of the rectus femoris tendon respectively [67, 68]. Of note, it is speculated that using a local graft that requires wide capsulotomy and local harvesting may further increase hip instability, especially in BDH [20, 68, 71, 72••].

The capsule also plays an essential role in BDH and should be addressed during hip arthroscopy. The joint capsule acts as a non-dynamic stabilizer restricting the femoral head’s excessive translation and rotational movement [73••, 74, 75]. Most commonly performed hip arthroscopy involves capsulotomy to gain access to the hip joint, with the most common techniques being interportal and T-shaped capsulotomies [74, 76, 77]. Further, in hip dysplasia, the capsule is generally lax and redundant due to the bony undercoverage and ongoing instability [3•, 9, 73••, 74, 78]. In a cadaveric study by Johannsen et al. [72••], it was shown that the anterior hip capsule plays a vital role in controlling hip rotation and femoral head displacement. This finding correlates to the importance of the iliofemoral ligament (IFL) which comprises the anterior hip capsule and is considered to be the most essential ligament among the rest of the capsular ligaments [73••]. Additionally, interportal capsulotomy requires cutting of the iliofemoral ligament, exposing the hip to instability [75]. Weber et al. [79] examined different types of interportal capsulotomies and concluded that the disruption of the IFL is significantly smaller when the capsulotomy is performed between the anterolateral portal and the modified anterior portal, instead of the standard anterior portal [79].

Therefore, careful capsular management is essential, especially in BDH. The initial goal should be to avoid capsulotomies (other than for portal/instrument placement). If the technique requires capsulotomy to achieve surgical visualization and treatment, limiting the size of the capsulotomies should be considered. If a capsulotomy is required, recent studies recommend complete closure, or capsular plication, with a strong tendency toward capsular plication [1•, 3•]. Even with non-interportal or T capsulotomy procedures, capsular plication may be recommended to address capsular laxity. During the capsular plication procedure, the capsule is reshaped and tightened by creating an inferior shift of the capsule to augment the screw-home mechanism of the capsuloligamentous structures to provide more stability [3•, 80]. In a systematic review by Kuroda and colleagues [3•], most studies recommended performing capsular plication according to the suture shuttle technique described by Domb et al. [80]. Cvetanovich et al. [81] assessed the differences in the outcomes of hip arthroscopic surgery for patients suffering from FAI with and without BDH. Their results showed that capsular plication yielded significant clinical improvements regardless of whether the acetabulum had normal coverage or BDH. Kalisvaart et al. [82] performed a different type of capsular tightening procedure—the RICH procedure.

Laterally, the capsule has an autonomous area with no capsular reinforcement. This area is between the iliofemoral and ischiofemoral ligaments, or an area analogous to the rotator interval of the shoulder. In this procedure, an area of the lateral capsule is excised, usually 6–8 mm from proximal to distal, and 15 mm from anterior to posterior. The capsule is then sutured back, resulting in a rotator interval closure of the hip (RICH). This does not directly tighten the iliofemoral ligament, but does remove redundancy from the iliofemoral and ischiofemoral ligaments [82]. The results demonstrated a significant improvement in the patients’ PROs after hip arthroscopy and capsular plication for hip microinstability—and the outcomes were the same whether subjects had BDH or normal acetabular anatomy. In summary, in BDH, the capsule and specifically the IFL should be spared when possible, capsular closure mandatory when capsulotomy is made, and capsular plication is recommended [82].

As previously discussed, cam deformity in the presence of BDH has a different impingement pattern than patients suffering from FAI without BDH [25]. Kobayashi et al. [25] used a 3-dimensional computer simulation to identify the distribution of the impingement region in cam-type FAI and BDH with cam-type FAI. The authors found a significant difference between the two groups, demonstrating a more proximal impingement point for the combination of FAI and BDH. This finding should be taken into account when performing osteochondroplasty at the femoral head and junction [25].

The key is identifying if impingement is occurring, and thus be addressed, or if the main pathology is microinstability alone. Regardless, maintaining labral tissue and careful management of the capsule are critical to the success of hip arthroscopy in the setting of BDH and FAI.

## Hip Arthroscopy—Complications and Limitations

As previously discussed, more recent short- and mid-term outcomes for hip arthroscopy look promising with a significant satisfaction rate and improvement in almost all the PROs [1•, 3•, 23, 58, 59, 61, 81, 83, 84]. However, hip arthroscopy is not free of complications. Fukui et al. [70] operated on 102 hips with FAI and BDH, and reported on their clinical outcomes. Of these, 7 hips required a revision hip arthroscopy, and 5 hips were converted to total hip arthroplasty at a mean of 2-year follow-up period. The author concluded that the subsequent procedures were similar to those in patients having solely FAI and labral repair [70]. Likewise, Nawabi et al. [24] compared 55 hips with BDH and FAI to a sex-matched control group of 152 hips solely with FAI. Both groups underwent cam decompression, labral repair, and capsular closure at a similar percentage rate, and showed significant improvement in all patient-reported outcome scores at a mean follow-up period of 2 years postoperatively. Further, multiple regression analysis showed no significant differences in the PROs between the two groups, with an almost identical rate of revision arthroscopy in the BDH and FAI groups compared to the control group (4.3% and 4.6%, respectively) [24]. Cvetanovich et al. [81] also assessed the differences in the outcomes of hip arthroscopic surgery for patients suffering from FAI with and without BDH (36 and 312 patients respectively), with their primary outcome measure being the Hip Outcome Score–Activities of Daily Living (HOS-ADL). Their results showed significant improvements in all the PROs at a 2-year follow-up period (i.e., HOS-ADL, mHHS, VAS, HOS-Sports, and patients Satisfaction). An interesting finding was that capsular plication yielded significant clinical improvements regardless of whether the acetabulum had normal coverage and there were no significant differences between the two groups in outcome scores [81]. Within the BDH group, female patients demonstrated greater improvements in the mean HOS-ADL compared with male patients. Nonetheless, the relatively small sample size of the BDH group, and the fact that 28 to 41% of patients in the BDH group did not meet the threshold of patient acceptable symptom state (PASS), may suggest that these patients have some unreported residual symptoms and should suggest that further investigation is necessary to truly determine if the outcomes are truly the same between the groups [62, 81]. Domb’s study group [1•, 3•, 23, 58, 59, 61, 83] reported a case series of 55 patients presenting both BDH and FAI. The patients were followed for 5 years postoperatively with a significant improvement in all the PROs. The average rate of revision hip arthroscopy was 7.3% (range 0.9–15.6%), and the mean total revision rate was 11.1% (range 1.8–30.0%), with 2.1% of conversion to total hip replacement (THR).

Larson et al. [85•] reported on 88 hips with dysplastic radiographic findings and an age-matched cohort without hip dysplasia, followed for a mean of 26 months after hip arthroscopy. Failure was defined as conversion to THR or pelvic/femoral osteotomy, and as mHHS ≤70. The failure rate among the dysplastic cohort was 32.2%, compared with 10.5% failures for the FAI cohort [85•]. Moreover, compared with the control group, the postoperative mHHS among the BDH group was nearly 7 points less (88.4 vs 81.3, respectively), with 81.2% good/excellent results in the control group compared with the dysplastic group [85•]. One reason for these unfavorable results may be attributed to the fact that these patients had a full spectrum of hip dysplasia, not just borderline. Larson’s subjects had a mean LCEA and mean Tӧnnis angle in the dysplastic group of 20.8° and 11.0° respectively, with a range of the LCEA and Tӧnnis angle between 8.7° and 22.2° and between 0° and 22.2° respectively. A recent systematic review [3•] reported a total mean complication rate of 1% ranging from 0 to 4.8%, with the most prevalent complications being residual numbness and wound infection treated orally with antibiotics.

The most common cause of arthroscopic failure and complications is inappropriate patient selection. As discussed above, most of the studies define the lower limit of LCEA for BDH as 20°, with some studies defining it as 18° [1•, 3•, 6, 11]. An angle below this value is considered overt dysplasia. In a systematic review by Shah et al. [6] which included 773 dysplastic hips treated arthroscopically, the average failure rate was 25.8% at an average of 28.1-month follow-up. The review included thirteen studies with various degrees of dysplasia with a mean preoperative LCEA of 20.6°. The main radiographic predictors of failure were LCEA < 15°, Tönnis angle > 20°, broken Shenton line, and decreased joint space of ≤ 2 mm, as well as severe cartilaginous lesions [6]. It was shown that hip arthroscopy is more beneficial and effective in treating borderline-to-mildly dysplastic. Likewise, Hatakeyama and colleagues [22] reported that age ≥ 42 years old, broken Shenton line, overt osteoarthritis, Tönnis angle ≥15°, and vertical center anterior (VCA) angle ≤ 17° on preoperative radiographs are predictors of poorer outcomes from hip arthroscopy, but again, this suggests that overt dysplasia is likely best treated with non-arthroscopic means. In a match-controlled study by Chaharbakhshi et al. [86], patients with BDH (LCEA 18–25°) and excessive femoral anteversion (EFA) ≥20° were prospectively followed for 2 years after surgery. Though all the patients showed significant improvements after hip arthroscopy, the combined BDH and EFA group yielded significantly inferior results compared to the control group. The authors concluded that PAO or femoral osteotomy might be preferred in this case. Moreover, due to the excessive femoral and acetabular anteversion, the femoral head is practically exposed, which results in “functional” undercoverage [1•, 86].

Larson et al. [85•] reported that combining capsular plication and labral repair in a dysplastic hip achieved good to excellent results, as well as lower failure rates compared with the residual dysplastic cohort which was not treated with this combination. Grade 4 chondral defects were predictive of lower scores [85•]. In a review article by Kalisvaart et al. [9] discussing hip microinstability, the authors defined microinstability as an extraphysiologic hip motion accompanied by pain and additional symptoms. This phenomenon shares similar characteristics to hip dysplasia, which might manifest as instability. The authors suggested that in symptomatic patients who failed non-operative modalities, surgical soft tissue balancing focusing on the capsuloligamentous complex should be considered first. Other studies attributed hip arthroscopy failure to hip instability due to ligamentum teres (LT) tear, iliopsoas tenotomy in patients with psoas tendinitis or coxa saltans. It was speculated that BDH and other causes of instability may lead to inferior outcomes or failure after hip arthroscopy [1•, 83, 87, 88].

Thus, based on more recent literature where capsular management and differentiation between FAI and BDH have come to the forefront, outcomes of hip arthroscopy in the context of BDH demonstrate nearly equivalent results compared with FAI without BDH.

## Hip Arthroscopy and Periacetabular Osteotomy (PAO)

With traditional PAO, where the joint was not entered, and thus labral pathology not addressed, long-term outcomes were in the 60–85% success range at 10- to 20-year follow-up for hip dysplasia [85•, 89]. Some think that addressing the labrum through arthroscopy may improve these outcomes [3•, 90–94, 95•]. The combination of these two modalities can intensify the effect of hip preservation surgery, preserving more normal anatomical structures, facilitating the healing process, and potentially even delaying or preventing the need for future THR [3•, 90–94, 95•]. Domb et al. [90] performed hip arthroscopy to evaluate and address concomitant intra-articular pathologies prior to PAO, and noted several advantages. The first advantage is direct visualization of the entire joint allowing for inspection of the intra-articular pathology such as labral tears, cartilage flaps, and chondromalacia, as these findings may influence the surgeon’s decision regarding the desired procedure. Secondly, the surgeon can treat these pathologies before the PAO [90]. Greater joint visualization using the arthroscope compared to anterior arthrotomy can allow the surgeon to address a wider range of hip pathologies and provide more complete treatment. Hartig-Andreasen et al. [91] followed 95 hips that underwent PAO as the treatment for acetabular dysplasia for a minimum of 2 years postoperatively. The authors identified several risk factors predicting the need for a hip arthroscopy after PAO, such as acetabular retroversion (i.e., crossover sign), complete labral detachment, and preoperative BDH (LCEA 20–25°). Interestingly, labral degeneration, tearing, or hypertrophy did not negatively affect the outcome of PAO [91]. A large prospective case series by Sabbag and colleagues [95•] studied 248 hips in 240 patients who underwent combined hip arthroscopy and PAO. Seventeen patients had minor complications, and only 7 patients (3%) had major complications such as deep infection, wound dehiscence, and symptomatic heterotopic ossification. The overall survivorship of this group was 90% at 2 years and 86% at 3 years. Increasing age and diagnosis of acetabular retroversion were associated with higher complication and reoperation rates (hazard ratio 2.5 and 3.05 respectively) [95•].

Other studies investigating the necessity for revision hip preservation surgery after initial hip arthroscopy found that residual structural deformity such as in FAI and BDH was the leading cause [96, 97].

## Conclusions

Hip arthroscopy for BDH is an evolving procedure with promising short- and mid-term outcomes. Important factors include the degree of dysplasia and appropriate capsular management. By most accounts, mild or borderline hip dysplasia with LCEA between 20 and 25° and capsular plication are considered the best predictors for good arthroscopic outcomes. The combination of BDH and FAI is becoming recognized as a problem in its own right, requiring dedicated treatment. Further studies assessing long-term outcomes and indications for arthroscopic treatment of BDH and FAI are needed.
